# Construction of a ceRNA Network and Comprehensive Analysis of lncRNA in Hepatocellular Carcinoma

**DOI:** 10.3390/genes13050785

**Published:** 2022-04-28

**Authors:** Lin Wang, Jun Zhao, Cancan Zhu, Ke Yang, Ling Zhu, Yong Liu

**Affiliations:** 1 Anhui Institute of Optics and Fine Mechanics, Hefei Institutes of Physical Science, Chinese Academy of Sciences, Hefei 230031, China; wanglin3@mail.ustc.edu.cn (L.W.); zhaojun@aiofm.ac.cn (J.Z.); zhucancan@aiofm.ac.cn (C.Z.); keyang@aiofm.ac.cn (K.Y.); zhul@aiofm.ac.cn (L.Z.); 2 Science Island Branch of Graduate School, University of Science and Technology of China, Hefei 230026, China

**Keywords:** hepatocellular carcinoma, competing endogenous RNA, mRNA–miRNA–lncRNA, bioinformatics analysis

## Abstract

To explore the RNA biomolecular marker associated with hepatocellular carcinoma (HCC) prognosis, we constructed a regulatory network of competitive endogenous RNAs (ceRNAs), which provides favorable conditions for the early diagnosis, prognostic monitoring, and personalized treatment of HCC. In this study, the differentially expressed genes (DEGs) of patients with HCC were obtained from the Gene Expression Omnibus. We identified 574 upregulated genes and 274 downregulated genes relevant to HCC occurrence (*p* < 0.05). Subsequently, we constructed the protein–protein interaction (PPI) network using these DEGs and identified the hub genes from the PPI. We then determined the expression and prognostic values of the hub genes from the GEPIA and Kaplan–Meier plotter databases. After the upstream microRNAs (miRNAs) and long non-coding RNAs (lncRNAs) were respectively identified by miRTarBase and miRNet, we validated the expression of the key miRNAs in the serum using qPCR experiments. Moreover, we identified a two-lncRNA (LINC01184 and ADORA2A-AS1) signature from the upstream lncRNA that effectively predicted overall survival and had promotive effects for HCC. To verify the clinical significance of the signature, we validated the expression of the lncRNA in HCC tissues. Finally, we discovered and identified four mRNAs, four miRNAs, and five lncRNAs associated with the prognosis of HCC and constructed a new ceRNA regulatory network, which will be beneficial for the accurate diagnosis and treatment of HCC.

## 1. Introduction

Hepatocellular carcinoma (HCC) is a widespread malignancy that causes a serious threat to public health. Among all cancers, liver cancer ranks fifth in terms of global incidence and second in mortality among men [1]. Due to the insidious onset rapid progression of HCC, only 30–40% of patients are diagnosed at an initial stage and are available for curative-intent treatments such as surgery, ablation, or liver transplantation [2]. Meanwhile, HCC is highly aggressive and metastatic, leading to a poor prognosis and a high risk of postoperative recurrence. Although aggressive therapy measures are used, patients with advanced HCC still have a poor prognosis [3]. Therefore, the prevention and treatment situation of liver cancer is challenging.

In 2011, Salmena et al. first introduced the hypothesis of competing for endogenous RNA (ceRNA) [4]. It was shown that mRNA and common non-coding RNAs, such as lncRNA [5], pseudogene [6], and circRNA [7], could participate in post-transcriptional regulation of protein expression by competitively binding miRNA response elements and forming a complex regulatory network. If the expression of RNA-like genes is disturbed, the balance of the regulatory network can be disrupted and lead to the development of diseases [8]. There is growing evidence that the ceRNA regulatory networks are closely related to many types of cancer [9,10]. For example, the lncHILAR promotes the invasion and metastasis of renal cell carcinoma through miR-613/206/1-1-3p [11]. The tumorigenic lncRNA ITGB8-AS1, which is strongly upregulated in colorectal carcinoma, functions as a ceRNA that regulates focal adhesion signaling [12]. TUG1 is an oncogene that regulates *SRSF9* expression through miR-328-3p, promoting liver cancer proliferation and metastasis [13]. Using the ceRNA network, we can identify undetected biomarkers that could lead to an earlier diagnosis and treatment of cancer. Many articles have used the approach of obtaining DElncRNAs, DEmiRNAs, and DEmRNAs to build a ceRNA network, then taking intersections with miRNA–mRNAs and miRNA–lncRNAs [14,15,16,17]. This approach results in huge networks. In this paper, we use a “mRNA–miRNA–lncRNA” order model with a survival analysis in each step, which makes this ceRNA network more streamlined and correlates with the overall survival of patients.

**Figure 1:**
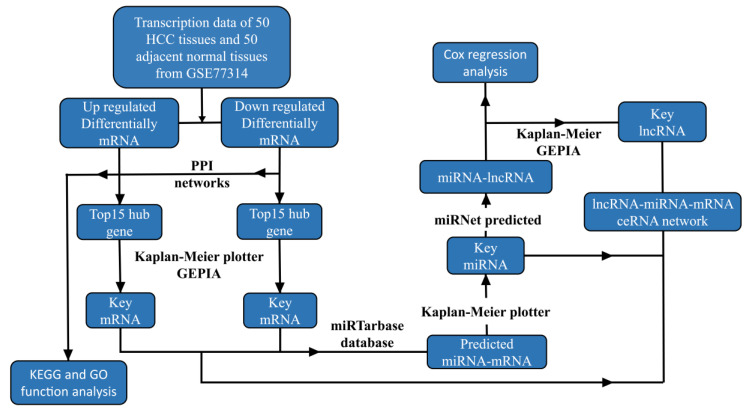
The workflow of ceRNA network study in HCC.

Above all, in this study, we screened and identified RNA-like markers related to HCC prognosis and investigated the molecular regulatory mechanisms of these markers in the ceRNA network. We finally constructed a novel prognosis-related ceRNA regulatory network for hepatocellular carcinoma. The workflow diagram is shown in Figure 1. The network provides new clues for preoperative precision diagnosis and postoperative recurrence monitoring in hepatocellular carcinoma.

## 2. Materials and Methods

### 2.1. Screening of the Original Data

The RNA sequencing data containing hepatocellular carcinoma (HCC) tissue and its adjacent normal tissue were searched from the Gene Expression Omnibus database (GEO). Only the GEO datasets with more than 30 samples were adopted. Then, we screened the titles and summaries of these datasets and further evaluated the complete information of the datasets. Finally, only the dataset GSE77314 sequenced by the Illumina Genome Analyzer (GPL9052) platform was selected for the follow-up study. The GSE77314 included 50 hepatocellular carcinoma samples and 50 normal tissue adjacent to carcinoma samples.

### 2.2. Screening of Differential Genes

We download the expression matrix from the GSE77314 dataset. We used CPM > 1 as cut-off values to filter out low expressed genes. Then, the data was calculated by the “edgeR” in R language to identify the DE mRNAs between HCC tissue and normal tissue. We calculated each mRNA expression difference ratio fold change (FC), set selection criteria: the log|FC| > 2, *p* < 0.05.

### 2.3. Functional Enrichment Analysis of DE mRNA

To obtain a deeper insight into the mechanism of the DE mRNAs in the development of LIHC, we use the “clusterProfiler package” in the R software for functional annotation analyses. The functions of the DE mRNAs were categorized into three categories: biological processes (BP), cellular components (CC), and molecular functions (MF).

### 2.4. Construction of PPI Network and Screening of Key mRNAs

The STRING database is used to explore the DEG and establish the protein–protein interaction (PPI) network. With the confidence score set to 0.4, we obtained the PPI networks of upregulated DE mRNAs and downregulated DE mRNAs. Then, we use the Cytoscape (version 3.6.1) to create a visualization PPI network. The MNC (maximum domain component) method in the Cytohubba plug-in was used for node analytics of the PPI network. We select the top 15 highest scores of the hub genes among upregulated and downregulated genes for subsequent analysis, respectively.

### 2.5. Survival Analysis

In HCC, we identified the prognostic values of all the RNA in ceRNA networks using the Kaplan–Meier plotter database [18]. Using clinical data from the Kaplan–Meier plotter database to analyze key mRNAs, miRNAs, lncRNAs, and plot survival curves. The log-rank *p*-value and hazard ratio (HR) was then automatically computed and presented. When the log-rank *p*-value < 0.05, the survival analysis was regarded as statistically significant.

### 2.6. Gene Expression Analysis

Gene Expression Profiling Interactive Analysis (GEPIA) contains the RNA sequencing expression data from the TCGA and the GTEx projects [19]. We used the GEPIA database to calculate the expression levels of key mRNAs and lncRNAs in HCC. These key RNAs with |log2FC| > 1.5 and *p*-value < 0.05 were considered as statistically significant.

### 2.7. Construction of ceRNA Network

The miRTarbase tool was used to forecast the miRNAs that interacted with the key mRNAs. LncRNAs that targeted the key miRNAs were forecasted by the miRNet database. Cytoscape software (version 3.6.1) was used to construct and map the three-level ceRNA regulatory network of mRNAs, miRNA, and lncRNAs.

### 2.8. Tissue Samples and Serum Collection

We collected blood samples from two groups of participants: 40 patients diagnosed with HCC and 20 healthy volunteers (Table 1). Any patient with a history of cancer was excluded, except those with HCC, and none of the healthy controls displayed any symptoms of liver disease. We obtained nine pairs of HCC tissues and their adjacent normal tissues from the First Affiliated Hospital of the University of Science and Technology of China (Table 2). All the HCC patients have been pathologically confirmed and immediately stored in RNAlater solution before use.

### 2.9. RNA Expression Analyses

The total RNA and miRNA were extracted by TRIzol reagent (Invitrogen, Carlsbad, CA, USA) and miRNeasy Serum/Plasma Kit (Qiagen, Hilden, NRW, Germany) according to the manufacturer’s instructions. The reverse-transcribed total RNA was using PrimeScript reverse transcription reagent (Takara, Otsu, Shiga, Japan) or Mir-X™ miRNA First-Strand Synthesis Kit (Takara, Japan). Quantitative PCR analysis of lncRNA LINC01184 and GAPDH was carried out using the LightCycler 480 instrument with TB Green Premix Ex Taq II (Takara, Japan). Quantitative PCR analysis of key miRNAs and miR-4644 was performed using TB Green Advantage qPCR Premix (Takara, Japan) according to the manufacturer’s protocol. The experiment was repeated three times for each sample, and the RNA expression was computed according to the 2^−ΔΔCt^ approach. The primer sequences used in this study are shown in Table S2.

### 2.10. Statistical Analysis

We used an unpaired *t*-test to complete the differential expression analysis of key miRNAs and lncRNAs. We used the GraphPad Prism 9.0.0 to calculate the correlation between lncRNA–miRNA–mRNA expression. The COX regression screening of lncRNAs using the “survival” in R language. We considered it statistically significant at *p* < 0.05.

## 3. Results

### 3.1. Screening of Differential Genes for Liver Cancer

**Figure 2:**
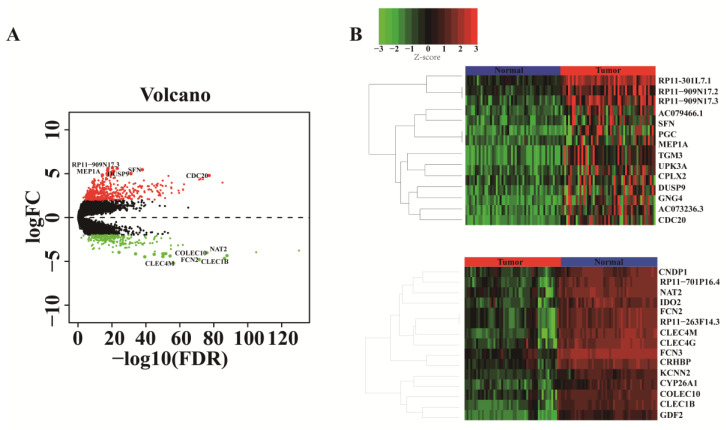
Identification of DEmRNAs in HCC. (**A**) Volcano plot showing the DEmRNAs identified from GSE77314. The points become larger in the top 15 up- and downregulated genes, the top five up- and downregulated gene names are labeled in the volcano. |log2FC| ≥ 2 and a *p*-value ≤ 0.01 were set as the truncation criteria. (**B**) Heatmap of top 15 up- and downregulated DEmRNAs.

We analyzed DEmRNAs between 50 HCC and 50 corresponding paracancerous samples with |logFC| ≥ 2 and a *p*-value ≤ 0.01. We identified 574 upregulated genes and 274 downregulated genes relevant to HCC. The volcano plots illustrate the distribution of DEmRNAs (Figure 2A), and the heatmap shows the top 15 upregulated and downregulated DEmRNAs (Figure 2B). The top 15 DEmRNAs with their log2FC values and *p*-values are shown in Table S1.

### 3.2. Functional Enrichment Analysis of DEmRNAs

**Figure 3:**
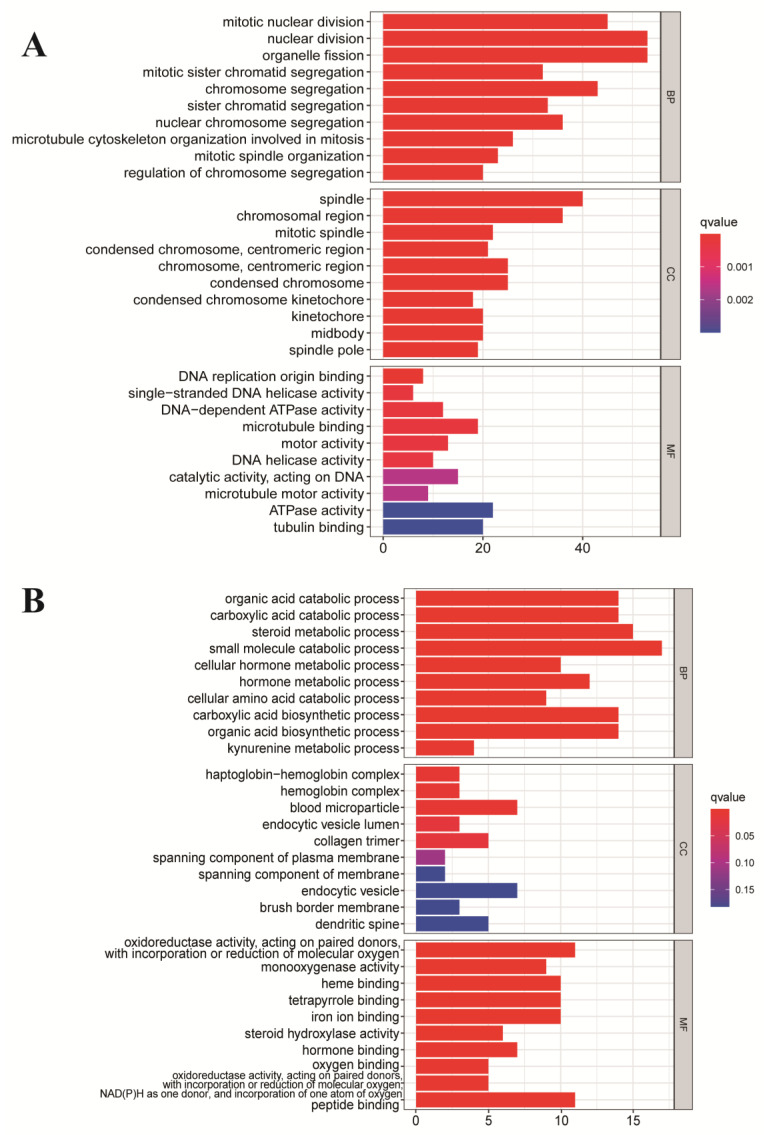
GO functional annotation for the DE mRNAs. (**A**) Upregulated DE mRNAs. (**B**) Downregulated DE mRNAs.

In order to analyze the potential molecular mechanism of these genes, we conducted the GO function enrichment analysis for DE mRNAs. The three distinct categories of GO function enrichment analysis are shown in Figure 3. The enriched GO functions for the upregulated DE mRNAs included mitotic nuclear division, nuclear division, organelle fission in the BP category; ATPase, tubulin binding, and microtubule-binding in the MF category; spindle, chromosomal region, and chromosome in the CC category (Figure 3A). The results indicate that the majority of the upregulated genes were related to cell division behavior, and we believe that they are involved in the vital activities of HCC development.

The GO functions enriched by the downregulated DEGs are shown in Figure 3B, including small molecule catabolic, steroid metabolic, the organic acid biosynthetic process in BP category; blood microparticle, endocytic vesicle, collagen trimer in the CC category; oxidoreductase activity, acting on paired donors in MF category. The functions in the downregulated genes are related to metabolism and redox reactions, which are also relevant to the activity of cancer cells, but it can be seen that the number of genes enriched for each function in the downregulated genes is smaller and the results for the upregulated genes are more plausible.

### 3.3. Establishing the PPI Network and Screening of Hub Genes

**Figure 4:**
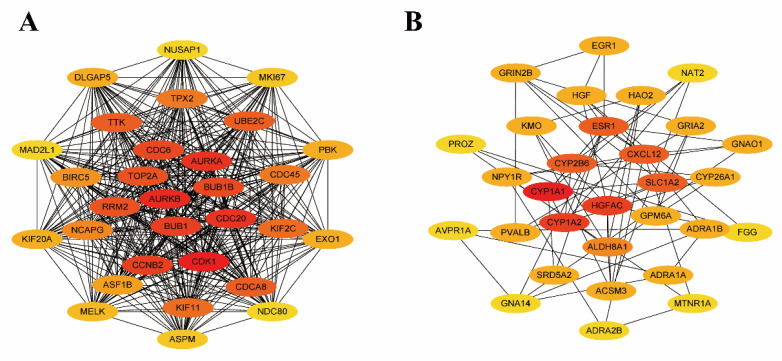
The top 30 hub genes were identified from DE mRNAs. The redder the color, the higher the ranking. (**A**) Visualization of the top 30 upregulated hub DE mRNA. (**B**) Visualization of the top 30 downregulated hub DE mRNA.

Using the PPI network to predict cancer-related Hub genes is an effective strategy. The STRING database analyzed the DEmRNAs and constructed the upregulated genes and downregulated genes as the PPI network, respectively. According to the maximum neighborhood component (MNC), the top 30 hub genes were screened from the DEmRNAs by the Cytoscape software. Figure 4 shows the interaction of the top 30 hub genes for upregulated and downregulated genes.

**Figure 5:**
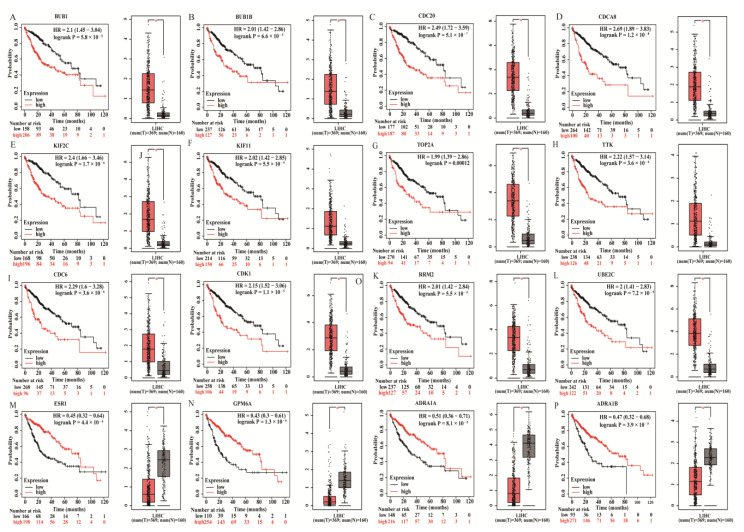
Screening the key genes in HCC. (**A**–**L**) Expression and prognostic values of *BUB1*, *BUB1B*, *CDC20*, *CDCA8*, *KIF2C*, *KIF11*, *TOP2A*, *TTK*, *CDC6*, *CDK1*, *RRM2*, and *UBE2C* in HCC. (**M**–**P**) Expression and prognostic values of *ESR1*, *GPM6A*, *ADRA1A*, and *ADRA1B*.

We investigated the relationship between the DEmRNAs and HCC by determining the expression and prognostic values of the hub genes from the GEPIA database and Kaplan–Meier plotter databases, respectively. Finally, we found 12 upregulated genes (*BUB1*, *BUB1B*, *CDC20*, *CDCA8*, *KIF2C*, *KIF11*, *TOP2A*, *TTK*, *CDC6*, *CDK1*, *RRM2*, and *UBE2C*) were associated with a poor prognosis of HCC when they are overexpressed (Figure 5). We also found that four downregulated genes (*ESR1*, *GPM6A*, *ADRA1A*, and *ADRA1B*) were associated with the favorable prognosis of HCC when they are overexpressed (Figure 5).

### 3.4. Integrated Analysis of miRNAs in the ceRNA Network

In order to identify miRNAs interacting with key genes, 16 key genes were analyzed by the miRTarBase database, and we finally predicted 366 miRNAs. We then analyzed the relationship between these 366 miRNAs and the overall survival of HCC patients by the Kaplan–Meier plotter tool.

**Figure 6:**
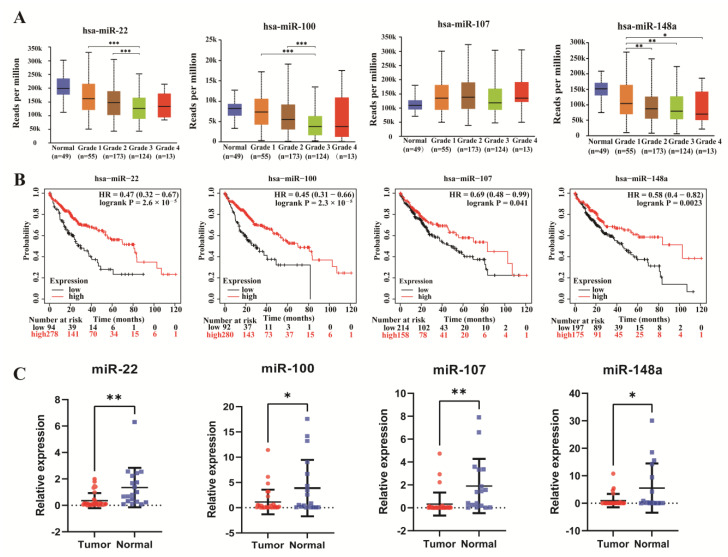
Comprehensive analysis of the key miRNAs (**A**) The miRNA expression with tumor grade in HCC. (**B**) Survival analysis of miR-22, miR-100, miR-107, and miR-148a in 371 HCC tissues. (**C**) Expression patterns of miR-22 (*p* = 0.0012), miR-100 (*p* = 0.0203), miR-107 (*p* = 0.0018), and miR-148a (*p* = 0.0154) in HCC and health groups (HCC: *n* = 40; healthy: *n* = 20). Data are shown as means ± SD, * *p* < 0.05, ** *p* < 0.01, *** *p* < 0.001.

Then, through the Kaplan–Meier survival curve, we obtained eight miRNAs associated with the OS of HCC. These eight miRNAs potentially regulate eight upregulated and one downregulated mRNA. These mRNAs targeted to the eight miRNAs are shown in Table S3. Based on the ceRNA hypothesis, these miRNAs should theoretically have opposite prognostic significance to targeted mRNAs. Survival analysis results showed that four of the eight miRNAs (MiR-22, MiR-100, MiR-107, and MiR-148a) were positive prognostic biomarkers of HCC patients (Figure 6B), of which prognostic effects were opposite to the key mRNAs targeted by them.

For further validation, the qPCR was used to test the serum of 40 HCC patients and 20 normal individuals with HCC patients. It was demonstrated experimentally that these four miRNAs were significantly downregulated in the serum of HCC patients (Figure 6C). Surprisingly, the expression of miRNAs (miR-22, miR-100, miR-148a) decreased with tumor grade advanced in four key miRNAs(Figure 6A).

### 3.5. Integrated Analysis of lncRNAs in the ceRNA Network

**Figure 7:**
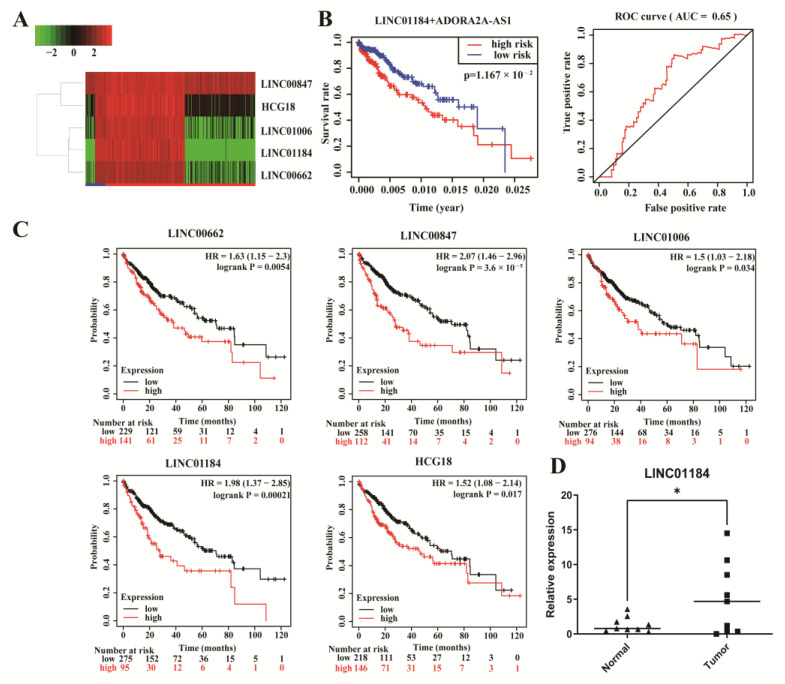
Comprehensive analysis for the 133 lncRNAs in HCC (**A**) Heatmap of the 5 key lncRNAs expression. (**B**) Kaplan–Meier survival analysis and ROC curve validation of two lncRNA signatures based on risk scores. (**C**) Kaplan–Meier survival analysis of five lncRNAs. (**D**) Expression patterns of LINC01184 in HCC and paracancerous tissues (Normal: *n* = 9; Tumor: *n* = 9). Data are shown as means ± SD, * *p* < 0.05.

To further obtain lncRNAs that bind competitively to key genes, we predicted those lncRNAs that have the potential to bind to the four miRNAs through the miRNet database. Then, a total of 133 lncRNAs were discovered. Based on the ceRNA hypothesis, there is a negative correlation between lncRNAs and miRNAs. Therefore, we used the TCGA dataset to determine the expression levels and prognostic values of key lncRNAs. The result is shown that (Figure 7), a total of five lncRNAs (HCG18, LINC00662, LINC00847, LINC01006, LINC01184) were identified with significantly higher expression (*p* < 0.05) in HCC, and they were also poor prognostic factors for OS.

Among the 133 predicted lncRNAs, we computed the risk scores of these lncRNAs by univariate and multivariate Cox regression analysis. Then, we constructed a prognostic index for HCC samples calculated by the formula: prognostic index (PI) = (0.2821 × expression level of LINC01184) + (−0.3049 × expression level of ADORA2A-AS1). Patients were further classified according to risk scores, and the predictive power of this predictive model was verified by the Kaplan–Meier survival curves (*p* = 0.01167) and receiver operating characteristic (ROC) curve analysis (AUC = 0.65). Remarkably, LINC01184 was found to be a promotive factor for HCC with high prognostic diagnostic values. As further verification of this finding, LINC01184 was expressed higher (*p* < 0.05) in HCC tissues (*n* = 9) than in adjacent normal tissues (*n* = 9).

### 3.6. Construction of ceRNA Sub-Network

**Figure 8:**
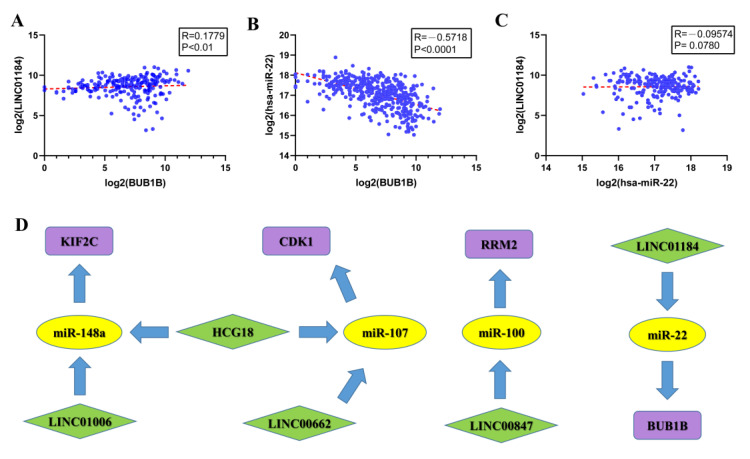
(**A**) Correlation between *BUB1B* and LINC01184. (**B**,**C**) Correlation between miR-22 and *BUB1B* and LINC01184. (**D**) Integration of the identified ceRNA network. Green diamonds stand for lncRNAs, purple rectangles stand for mRNAs, and yellow ellipses stand for miRNAs.

We constructed a lncRNA–miRNA–mRNA–sRNA competitive endogenous RNA network by a series of analyses, which only included LINC01184, miR-22, and *BUB1B*. According to the ceRNA hypothesis, the binding of lncRNA to miRNA can relieve the suppressive effect of miRNA on mRNA. Based on the theory, lncRNAs and mRNAs have inverse relationships between miRNA; mRNAs and lncRNAs have positive relationships. According to Figure 8B,C, miR-22 was found to be negatively regulated with lncRNAs and mRNAs, respectively, by Spearman analysis. We analyzed LINC01184 and *BUB1B* to find that the LINC01184 were positively correlated with mRNA (Figure 8A). In the future, these sub-networks may also serve as therapeutic targets or diagnostic biomarkers for HCC.

## 4. Discussion

The ceRNA hypothesis suggests that all types of RNA transcripts can form a large-scale regulatory network by competitively binding miRNA response elements. Meanwhile, disruption of the dynamic balance between ceRNAs and miRNAs can significantly affect the biological function of the organism, which in turn leads to cancer development. Several studies have shown that ceRNA networks have essential roles in the development of many tumors. However, ceRNA networks in hepatocellular carcinoma have not been comprehensively studied, and there are still many ceRNA molecules to be explored.

Therefore, we tried to screen the specific ceRNA molecules by mRNA–miRNA–lncRNA order patterns and identified the relationship between each RNA molecule and the prognosis of hepatocellular carcinoma. All RNAs in the novel ceRNA network showed significant prognostic values in HCC, which may provide alternatives to biomarkers and potential therapeutic targets.

Based on the 50 liver cancer samples from GEO dataset GSE77314, we obtained 848 significant DE mRNAs, which contained of 574 upregulated and 274 downregulated DE mRNAs. These DE mRNAs subjected to GO analysis showed that they were significantly enriched in some cancer biological behaviors, such as cell division, metabolism, etc. The majority of upregulated genes are engaged in cell mitosis, cell cycle and are necessary for cancer cells to continue to divide.

To systematically analyze the relationship and function of DE mRNAs in hepatocellular carcinoma, we constructed a PPI network. It can be seen that the interactions of upregulated genes are more concentrated. Generally, the DE mRNAs with more node degrees tend to have a more central role in the network. Therefore, we used CytoHubba to screen for hub genes in both up- and downregulated networks. For further identifying key genes in HCC, the Kaplan–Meier survival curve was plotted on the top 15 up and downregulated hub genes, and 16 key mRNAs were obtained. Twelve of the key mRNAs were upregulated, and four of them were downregulated; the results of the survival curve revealed that high expression of all upregulated key mRNAs was linked to poor prognosis of HCC, and high expression of all downregulated mRNAs was linked to good prognosis of HCC. For example, KIF2C is a substantial mitotic regulator that promotes HCC cell proliferation and hinders apoptosis. *KIF2C* was probably a novel target for the precise treatment of HCC [20]. CDK1 is a key G2/M checkpoint protein in the cell cycle that is used as an HCC therapy target [21,22,23]. RRM2 is a ribonucleotide reductase subunit, which is silenced can inhibit HCC proliferation [24]. In the results of GO enrichment analysis, the molecular function of RRM2 was “oxidoreductase activity, acting on CH or CH2 groups” and “ferric iron-binding”. Therefore, its high expression helps HCC cells to have more oxidoreductase activity, which promotes its proliferation. BUB1B is a mitotic checkpoint serine/threonine kinase B, which promotes HCC development by activating the mTORC1 signaling pathway [25]. In the results of GO enrichment analysis, the KIF2C, CDK1 and BUB1B are all enriched in the “nuclear division”, “mitotic nuclear division”, and “organelle fission”. These biological processes all represent cell division, so these three genes together regulate the division process of HCC cells.

As mentioned previously, miRNAs and lncRNAs regulate gene expression through the ceRNA mechanism. Therefore, we predicted the upstream miRNAs and explored key miRNAs from them by survival analysis. The results revealed that the high expression of four miRNAs (miR-148a, miR-107, miR-22, miR-100) was associated with a good prognosis in hepatocellular carcinoma. Meanwhile, we demonstrated that these four miRNAs were downregulated in the serum of HCC patients by the vitro experiments. Many studies have reported the role of these four miRNAs in cancer. For example, Han et al. suggested that plasma miR-148a is a possible biomarker for HCC screening [26]; miR-107 suppresses the proliferation of HCC cells by targeting *HMGA2* [27]; the dysregulation of miR-100 is an early event in HCC progression [28], and its inactivation contributes to hepatocarcinogenesis in vivo [29]. Meanwhile, miR-22 has an inhibitory effect on HCC, and its overexpression inhibits HCC cell growth, invasion, and metastasis both in vitro and in vivo [30]. Intriguingly, most of these key miRNAs have been studied in Hepatocellular carcinoma (HCC).

Several studies have indicated that LncRNAs play a critical role in cancer. Therefore, we predicted 133 lncRNAs targeting these four key miRNAs. Through performing expression and survival analysis of the lncRNAs in hepatocellular carcinoma, we found that five lncRNAs (LINC00662, HCG18, LINC00847, LINC01006, LINC01184) were confirmed as the key lncRNAs. Among the key lncRNAs, the LINC00662 contributes to oncogenic behaviors both in vitro and in vivo [31]. HCG18 is involved in vascular invasion of HCC through regulation of macrophages and tumor stem cells [32]. Silencing LINC01006 suppresses the viability of HCC cells in vitro and suppresses tumor growth in vivo [33]. The roles of LINC00847 and LINC01184 have not been reported in HCC, but several studies have demonstrated that they have a carcinogenic effect in many other cancers [34,35,36]. Thus, a novel mRNA–miRNA–lncRNA network has been established to be associated with the hepatocellular carcinoma prognosis. Several pairs have been verified in this ceRNA network. It was shown that LINC00662 promoted the growth and metastasis of hepatocellular carcinoma tumors through competitively binding miR-15a, miR-16, and miR-107 [37]. In addition, these reports confirm the accuracy of our current analytical results.

We then analyzed 133 upstream lncRNAs by computing risk scores with univariate and multivariate Cox regression analysis. The analysis revealed that LINC01184 and ADORA2A-AS1 were of significance in predicting the prognosis of HCC. However, only LINC01184 was involved in the establishment of a complete endogenous regulatory network. According to the comprehensive view, LINC01184 could serve as a prognostic and diagnostic biomarker for HCC. Furthermore, we verified that the LINC01184 was upregulated in HCC tissues. In this integrative research, we investigated a key ceRNA axis of the ceRNA network, including LINC01184/miR-22/BUB1B. Finally, we computed the correlation between the ceRNA in the mRNA–miRNA–lncRNA axis showed that the LINC01184/miR-22/BUB1B absolutely conformed to the ceRNA hypothesis. Actually, we have found some interesting results in our study through a series of bioinformatics analyses, but more cellular experiments and large-scale clinical studies are necessary in the future.

In addition, the GSE77314 database was once mined for the gene *DMGDH* [38], which inhibits metastasis in hepatocellular carcinoma, but the differential multiplicity of the gene did not meet the authors’ screening criteria; thus, it was filtered out. It shows that it is possible to screen the same database with different methods to find meaningful genes, and it is important to use as many different methods as possible to mine more different meaningful genes and phenomena.

## Figures and Tables

**Table 1:** The clinical features of patients in the serum samples recruited for this study.

Clinical Variable	Numbers (*n* = 40)	Percentage
**Age, *n***		
<60	21	52.5%
>60	19	47.5%
**Gender, *n***		
Female	16	40%
Male	24	60%
**ALT(IU/L), *n***		
Low (<50)	18	45%
High (>50)	22	55%
**AFP(ng/mL), *n***		
Low (<7)	13	32.5%
High (>7)	27	67.5%
**Tumor size, *n***		
Small (<3 cm)	13	32.5%
Large (>3 cm)	24	60%
NA	3	7.5%

**Table 2:** The clinical features of patients in the tissue samples recruited for this study.

Clinical Variable	Numbers (*n* = 9)	Percentage
**Age, *n***		
<60	7	77.8%
>60	2	22.2%
**Gender, *n***		
Female	1	11.1%
Male	8	88.9%
**Grade, *n***		
Grade 1	1	11.1%
Grade 2	3	33.3%
Grade 3	5	55.6%
Grade 4	0	0

## Data Availability

The data used in this study are from the GSE77314 datasets of the GEO database.

## Supplementary Materials

The following supporting information can be downloaded at: https://www.mdpi.com/article/10.3390/genes13050785/s1, Figure S1: The PPI network pictures.; Table S1: The Top 15 upregulated and downregulated differentially expressed mRNAs between tumor and normal tissues in HCC.; Table S2: Primers used in this study. Table S3: The mRNAs targeted to the eight miRNAs.
