# Supplementary material for: Construction of a ceRNA Network and Comprehensive Analysis of lncRNA in Hepatocellular Carcinoma
Source: Genes (Basel). 2022 Apr 28;13(5):785. doi: 10.3390/genes13050785 (PMC9141944; doi:10.3390/genes13050785)
Supplement: Supplementary file 1 [file genes-13-00785-s001.zip › genes-1665875-supplementary.pdf]

Table S1. The top 15 upregulated and downregulated DEmRNAs with their log2FC values and P-values.

Genes	logFC	logCPM	PValue
RP11-301L7.1	5.664809245	4.474145013	2.56E-22
RP11-909N17.3	5.586511692	4.400139912	2.80E-25
RP11-909N17.2	5.58651169	4.400139912	2.80E-25
AC079466.1	5.58453638	4.470629975	1.48E-19
SFN	5.448891744	5.14188419	1.13E-40
PGC	5.353119157	4.473454457	2.85E-19
MEP1A	5.290961895	4.012896379	3.70E-23
TGM3	5.192998419	4.544307211	4.83E-22
UPK3A	5.120539857	4.418173862	5.44E-22
CPLX2	4.990621414	4.362845781	5.31E-19
DUSP9	4.982415126	5.068947585	1.12E-33
GNG4	4.915973021	4.024800287	8.09E-22
AC073236.3	4.841904755	4.417417615	4.52E-16
DKK1	4.829706035	4.754734194	2.50E-19
CDC20	4.78672941	5.383990668	2.47E-81
CNDP1	-4.211023785	5.337137303	8.38E-48
RP11-701P16.4	-4.464106711	5.532701379	3.70E-53
NAT2	-4.35720821	5.921805802	5.65E-92
IDO2	-4.492025009	5.462556919	3.63E-42
FCN2	-4.802037343	5.690365256	3.90E-75
RP11-263F14.3	-4.802037422	5.690365256	3.90E-75
CLEC4M	-5.223075185	5.970627467	9.05E-60
CLEC4G	-3.985054141	6.076026818	2.15E-26
FCN3	-4.171762122	6.824659519	1.18E-53
CRHBP	-4.144477327	5.867305151	3.98E-53
KCNN2	-4.123260098	4.197185421	5.33E-55
CYP26A1	-4.155810072	4.435021946	1.61E-36
COLEC10	-4.031480792	4.830046023	6.91E-80
CLEC1B	-4.68008381	4.736052355	2.39E-90
GDF2	-4.400937357	3.817374684	1.67E-57

Table S2. Primers used in this study.

Name	Sequence (5'- 3')
miR-22-5p-F1	AACAAGAGTTCTTCAGTGGCAAGC
miR100-5p-F1:	AACAAGAACCCGTAGATCCGAACTT
miR-107-F1：	AACACGCAGCAGCATTGTACAG
miR148a-5p-F1：	AACAAGAAAGTTCTGAGACACTCCG
LINC01184-forward	GCAAGCGGTCTTCTCTGTCT
LINC01184-reverse	GTCTCCTGTTCGTGTCAGCA

Table S3. The mRNAs targeted to the 8 miRNAs

miRNA	Targeted mRNA
miR-22	BUB1B, ESR1
miR-100	ESR1, RRM2
miR-107	CDK1
miR-148a	KIF2C
miR-106a	RRM2, BUB1
miR-149	RRM2
miR-188	CDC20, CDCA8
miR-215	TTK

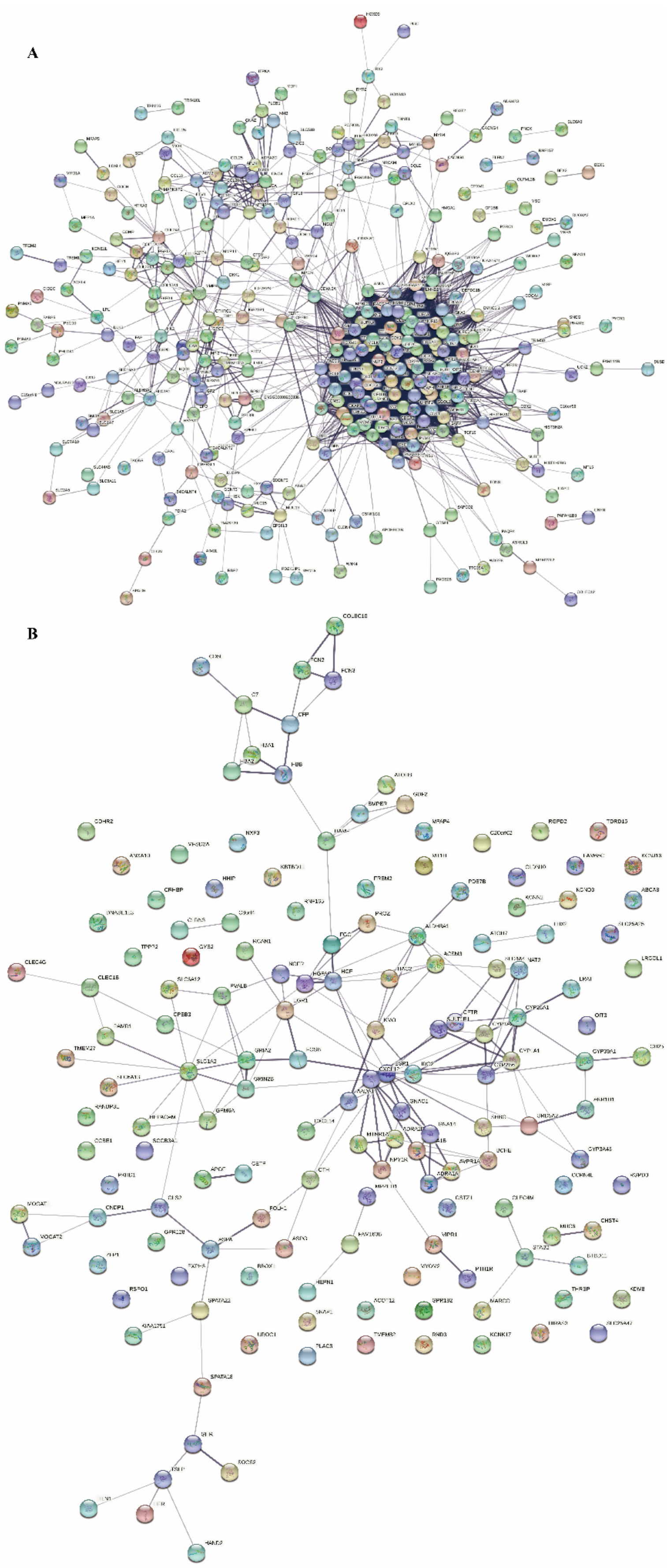

Figure S1. The PPI network pictures. (A) The PPI network of the significant upregulated DEGs.

(B) The PPI network of the significant downregulated DEGs.
